# The effects of light emitting diodes on mitochondrial function and cellular viability of M-1 cell and mouse CD1 brain cortex neurons

**DOI:** 10.1371/journal.pone.0306656

**Published:** 2024-08-30

**Authors:** Jong Soo Lee, Hyun Jin Park, Sang Ook Kang, Sang Hak Lee, Chang Kyu Lee

**Affiliations:** 1 Department of Ophthalmology, Pusan National University College of Medicine, Busan, Korea; 2 Biomedical Research Center, Ulsan University Hospital, University of Ulsan College of Medicine, Ulsan, Korea; 3 Department of Advanced Materials Chemistry, Korea University, Sejong, Korea; 4 Department of Chemistry, Pusan National University, Busan, Korea; 5 Department of Ophthalmology, Ulsan University Hospital, University of Ulsan College of Medicine, Ulsan, Korea; Massachusetts General Hospital, UNITED STATES OF AMERICA

## Abstract

The invention of Light Emitting Diode (LED) revolutionized energy-efficient illumination, but concerns persist regarding the potential harm of blue light to our eyes. In this study, we scrutinized the impact of LED light characteristics on eyes using two cell types: M-1 (rich in mitochondria) and CD-1 (neuronal). Variations in color rendering index (CRI) and correlated color temperature (CCT) were investigated, alongside exposure durations ranging from 0 to 24 hours. The findings illuminated the potential benefits of high-quality LED lighting, characterized by a high CRI and low CCT, which emits a greater proportion of red light. This form of lighting was associated with enhanced cell proliferation, elevated ATP levels, and reduced oxidative stress. In contrast, LEDs with low CRI and high CCT exhibited adverse effects, diminishing cell viability and increasing oxidative stress. These results suggest that high-quality LED lighting may have neuroprotective potential as a treatment option, such as for retinal ganglion cells.

## Introduction

Glaucoma stands as the second most prevalent cause of vision impairment globally [1, 2]. Central to its pathology is the progressive loss of retinal ganglion cells (RGCs) [3], spurring extensive research efforts aimed at unraveling the intricate mechanisms underlying RGC demise. Neurons, exemplified by Retinal Ganglion Cells (RGCs), stand out among diverse cell types for their susceptibility to mitochondrial impairment, owing to their heightened energy requirements and heightened sensitivity to reactive oxygen species (ROS) and apoptosis. A plethora of studies have underscored the correlation between mitochondrial dysfunction and a spectrum of neurodegenerative disorders, notably Parkinson’s disease and Alzheimer’s disease (AD), predominantly driven by oxidative stress [4–7]. Mitochondrial abnormalities serve as an initial catalyst for neuronal dysfunction, preceding observable degeneration in a glaucoma model [8].

Light can also affect mitochondrial function and the function of RGCs [9, 10]. Artificial lighting is fundamental to modern society, yet the emergence of advanced lighting technologies has raised concerns about the escalating health risks associated with light pollution [11]. Among these technologies, Light-emitting diodes (LEDs) emit heightened levels of blue light compared to traditional light sources, potentially exposing humans to unprecedented levels of blue light [12]. Given the environmental health implications, it’s imperative to evaluate the risks of retinal light injury and potential hazards stemming from prolonged exposure to LEDs before advancing further with this crucial, energy-efficient technology. Quality of LEDs, which may influence retinal damage, can be classified by color rendering index (CRI) and correlated color temperature (CCT), which are classic color metrics used to judge the quality of LED lights [12].

It’s hard to study the neuroprotective or damaging effect of different characteristics of LED light on RGCs, because the isolation of RGCs is known to be difficult and the commercial RGC-5 line has been withdrawn [13]. Moreover, pure RGCs are very vulnerable to certain environmental stimuli and are hard to maintain over a long period of time in a normal ambient environment. Therefore, we were going to study for similar cells like RGCs that could endure our study environment and could represent RGCs. Actually, RCGs have two basic characteristics: one is mitochondrial-rich and the other is neuronal cells. To carry out this study, we used a mouse kidney collecting duct cell, the M-1 cell, which is well-known as a mitochondrial-rich cell type [14], and mouse brain CD1 cortex neuronal cells, a neuronal cell line.

The main goal of this study was to evaluate the effects of different characteristics of LED light on similar cells like RGCs indirectly, by extrapolating from its effects on M-1 and CD1 cells; this was done in terms of functional and histological alterations induced by different LED light exposure, using the experimental methods described below.

## Materials and methods

### Cell preparation

M-1 (ATCC® CRL-2038™) cells were cultured in a nurturing blend of Dulbecco’s modified Eagle’s medium and Ham’s F12 medium, in a 1:1 ratio, enriched with 2.5 mM L-glutamine and carefully adjusted to maintain 15 mM HEPES, 0.5 mM sodium pyruvate, and 1.2 g/L sodium bicarbonate. The culture medium was further fortified with 0.005 mM dexamethasone and 5% fetal bovine serum to support cellular growth and viability. The cultures were maintained at 37°C in a humidified atmosphere comprising 5% CO2 and 95% air.

293T cells, originating from human embryonic kidney epithelial cells, were selected to juxtapose mitochondrial concentrations with those of M-1 cells. 293T cells were cultivated in Dulbecco’s modified Eagle’s medium supplemented with 2.5 mM L-glutamine, enriched with 15 mM HEPES, 0.5 mM sodium pyruvate, and 1.2 g/L sodium bicarbonate. This medium was further fortified with penicillin/streptomycin (Sigma, US) and 10% fetal bovine serum to sustain cellular growth and vitality. Subsequently, both cell lines were incubated under identical conditions, at 37°C in a humidified environment with 5% CO2 and 95% air. Mitochondria in M-1 and 293T cells were discerned utilizing Mito-ID green.

Mouse brain CD1 cortex neuronal cells (M-CX-400, Lonaz, Walkersville, MD 21793–0127, USA) were cultured in PNGMTM Primary Neuron Growth Medium BulletKitTM (CC-4461). The cultures were meticulously maintained at 37°C in a humidified atmosphere of 5% CO2 and 95% air.

Each vial of cortical cells contained an approximate count of 4 million viable cells. These cells were evenly distributed into 50 wells of a 96-well plate, adhering to recommended plating densities and utilizing the provided medium.

Within about 4 days of cultivation, the cells established a complex neurite network, with debris markedly reduced by the 7th day. Following the initial 2-hour media change post-seeding, further media changes were not advised until day 7. For extended cultivation, a 50% media renewal with fresh, pre-warmed medium was performed every 3 days thereafter.

Cellular assays were conducted after 12 days of sustained cultivation to assess their functional characteristics and responses under experimental conditions.

### LED lamp preparation

**Fig 1:**
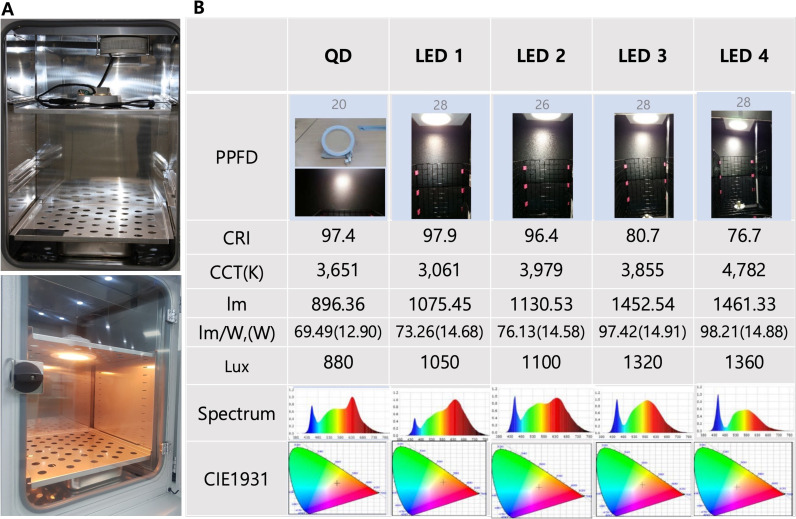
Values of several light emitting diodes (LEDs) used in this study. (A) A quantum dot (QD) LED was installed on the ceiling of a customized CO2 incubator. QD LED was turn on state (Botton) (B) Specifications of the LEDs which were used in this study. From LED1 to LED4, the CRI value decreased and the CCT value increased. Most values were similar in QD LED and LED1, but the distributions of the light spectra were different between the two LEDs. The QD LED spectrum was more focally distributed and contained a greater proportion of red waves than that of LED1. (QD: quantum dot LED, PPFD: photosynthetic photon flux density, CRI: color rendering index, CCT: correlated color temperature, Lux: Illuminance).

The experimental illuminating system included a 6 inch 15W down LED light and quantum dot bulb custom made by King Star Lighting (King Star, Incheon, Korea) characteristics of the bulbs are shown in Fig 1. As the bulbs went from LED 1 to LED 4, CRI values decreased and CCT values increased; the light spectrum and CIE 1931 color space were different at different settings. The major difference in lighting spectra from LED 1 to LED 4 was that the amount of red waves decreased and the amount of blue waves increased. The majority of values were similar between quantum dot (QD) and LED 1, however, the distribution of the lighting spectrum differed between the two LEDs. The spectrum of QD light was more focally distributed and contained a higher proportion of red wave, near-infrared, light, than did that of LED 1 (Fig 1B).

The experimental illumination system was situated at the top of the CO2 incubator and the temperature of the system was maintained at 37°C and humidity of system was also maintained over 96% to mimic cell culture conditions. The illumination system irradiated the basal surface of the plates (black) and was positioned 35 cm directly above the light source. After inserting the LED bulb and turning on the power in the incubator, the temperature inside the wells rose over the course of 30 minutes and then remained almost unchanged for the rest of the incubation time. The incubator was equipped with an auto cooling system (Vision Laboratory Instruments, Korea, www.visionbionex.com). Cells that were kept in the dark and incubated in the same incubator as the exposed cells were used as a control group. The exposure times were 0, 1, 1.5, 2, 4, 6 and 24 hours.

### Cell viability assay

#### 1) Trypan blue staining

5x105 cells per well were seeded onto a 6-well plate and allowed to incubate overnight. Cell viability was assessed using trypan blue dye exclusion method with a hemocytometer. Following trypsinization, cells were treated with a 0.4% trypan blue solution (Sigma) for 10 minutes, and more than 300 cells were scored under inverted microscopy. Both viable and nonviable cells were enumerated. The relative survival rate was calculated using the formula: Cell viability (%) = [OD (treatment groups)/OD (negative control)] X 100%.

#### 2) LDH assay

Cells were seeded into 96-well plates and permitted to adhere overnight. Following this initial incubation period, they were exposed to LED light for the appropriate durations. In triplicate wells, additional cells were plated to serve as spontaneous LDH activity controls (treated with water) and maximum LDH activity controls (treated with 10X Lysis Buffer). Fifty microliters of cell culture supernatant from each well were carefully transferred into a non-sterile, clear 96-well multiwell plate (Nunc, Thermo Fisher Scientific, Waltham, MA). To initiate the reaction, fifty microliters of reaction mixture from the Pierce LDH Cytotoxicity Assay kit (Thermo Fisher Scientific) were then added to each well. The plates were shielded from light and incubated at room temperature for 30 minutes to allow the reaction to proceed. Following incubation, the reaction was halted by adding 50 μL of stop solution.Quantification of LDH release was achieved by measuring absorbance at both 490 nm and 680 nm using a microplate reader. Subsequently, the obtained data were exported to Microsoft Excel (Microsoft Corp., Redmond, WA) for further processing. Normalization to the control condition (0 hours) was conducted before subsequent analysis in Prism 5.0 (GraphPad, La Jolla, CA). For the determination of LDH activity, the background absorbance at 680 nm was subtracted from the absorbance at 490 nm prior to the calculation of percent cytotoxicity. [(LDH at 490nm)—(LDH at 680nm)]. To determine percent cytotoxicity, we subtracted the LDH activity of the spontaneous LDH release control (water-treated) from the LDH activity of the chemical-treated sample. This difference was then divided by the total LDH activity. [(maximum LDH release control activity) (spontaneous LDH release control activity)], and the result was multiplied by 100:

%cytotoxicity=[(LED‐exposedLDHactivity–spontaneousLDHactivity)/(maximumLDHactivity‐spontaneousLDHactivity)]X100

### Mitochondrial function analysis

#### 1) WST-1 cell proliferation assays

The WST-1 assay capitalizes on the enzymatic activity of mitochondrial succinate-tetrazolium reductase, which catalyzes the cleavage of tetrazolium salts into a soluble formazan dye. This process occurs exclusively in viable cells and is directly correlated with cellular metabolic activity. Cells were initially seeded into 96-well plates, allowing them to adhere overnight, before being subjected to various light exposures. Subsequently, the WST-1 assay was conducted immediately after light exposure durations of 1, 1.5, 2, and 4 hours. Upon completion of light exposure, 10 μl of Cell Proliferation Reagent (www.donginls.com) was added to each well, followed by an incubation period of 0.5–1 hour at 37°C in darkness. After incubation, the resulting formazan dye was quantified by measuring its absorbance at 450 nm using a microplate reader.

#### 2) ATP assay

The colorimetric ATP assay was conducted according to the manufacturer’s instructions (BioVision, cat. # k354). In summary, 10 μl of both samples and standards were dispensed into 96-well plates preloaded with the ATP reaction mixture. Incubation was carried out at room temperature in darkness for 30 minutes.

Subsequently, the plates were subjected to reading using a microplate reader, measuring absorbance at 570 nm. All ATP assays were monitored for a duration of 4 hours.

#### 3) ROS measurement

ROS levels were quantified using BioVision’s ROS Detection Assay Kit. Cells were seeded into 96-well plates, with a recommendation for using a black plate with a clear bottom for optimal fluorescence measurement. Following overnight incubation to allow for adherence, the media was aspirated, and the cells were gently washed with 100 μl of ROS Assay Buffer. Subsequently, 100 μl of 1X ROS Label, diluted in ROS Assay Buffer, was added to each well, and the cells were incubated for 45 minutes at 37°C in darkness. To remove excess ROS Label, 100 μl of ROS Assay Buffer or PBS was added, and the cells were exposed to light for durations of 1, 1.5, 2, and 4 hours. The experimental setup included appropriate controls, along with blank wells containing only media or buffer. In one control experiment, cells were treated with 100 μM H2O2 for 4 hours prior to analysis.Fluorescence was then measured at Ex/Em = 495/529 nm in endpoint mode, in the presence of compounds and controls, to assess ROS levels accurately.

#### 4) TIRF (Total internal reflection fluorescence microscope) image

M-1 cells were exposed to control, QD LED, LED1, LED2, LED3 and LED 4 irradiation for 2 hours and Mito-green 530 were treated after LED exposure. Coverslips were carefully mounted onto the samples, facilitating the imaging process of Mito-green 530 fluorescence using a TIRF microscope. (lg-TIRFM, TIRF Labs, NC, USA). Three images of TIRF were analyzed: original image, color coded image and zoom in image [15]. 

### Statistical analysis

All data is expressed as mean ± SEM. Student’s t-test was used for two-group comparisons using Statistical Package for the Social Sciences (SPSS) for Windows (version 21.0, SPSS, Inc., Chicago, IL, USA). A P-value < 0.05 was considered statistically significant.

## Results

### Cellular viability differed according to type of LED exposure in M-1 cells

**Fig 2:**
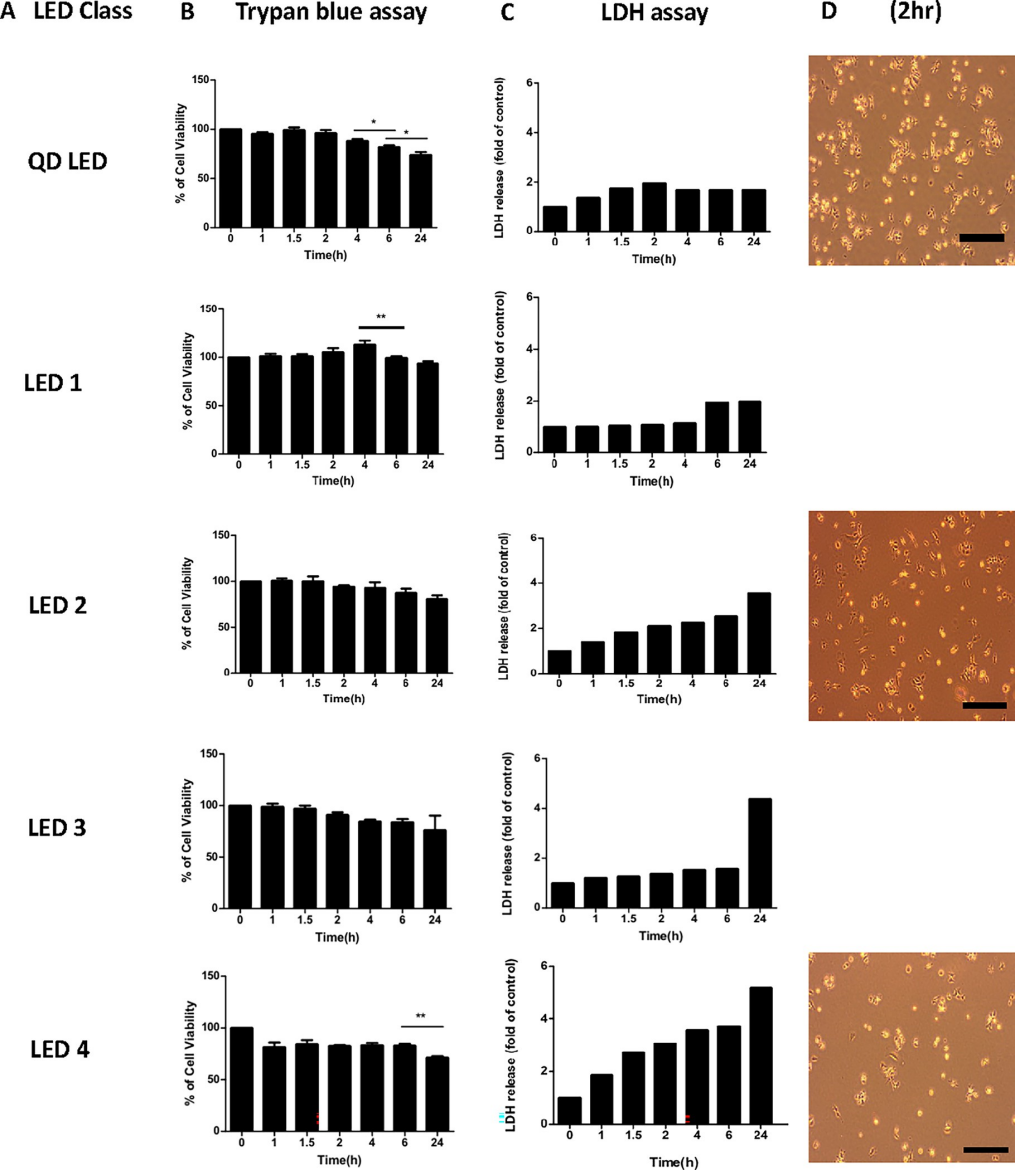
Cellular viability in M-1 cells according to type of LED and duration of LED lighting. The trypan blue assay and LDH assay were used in M-1 cells to assess cellular viability according to different classes of LED lights and different exposure times. Microscopic image of M-1 cells 2 hours after LED illumination with QD LED, LED 2, LED 4. Black scale bar: 100 μm, (* p < 0.05, **p < 0.01).

Cellular proliferation impairment and functional impairments of mitochondria were observed when M-1 cells were exposed to lower CRI and higher CCT LED light. This discovery was followed by an investigation into whether mitochondrial destruction caused cellular death. Therefore, we used trypan blue and LDH assays (Fig 2), which are widely used to detect cell death.

In the QD LED, LED1, and LED2 groups, cell viability was maintained for a certain period of time which corresponded to increased cellular proliferation and decreased ROS generation. In the LED3 and LED4 groups, cellular viability decreased after irradiation; viability was notably worse in the LED4 group (Fig 2).

The results of LDH assays revealed a similar reverse pattern as seen in the trypan blue assays (Fig 2). Like in trypan blue staining, LDH assays showed that higher CRI and lower CCT LED caused less cell death.

Microscope images of the QD LED, LED2, and LED4 groups were taken toreveal cell density, which indicates cellular viability, in the same area of the well in each group after 2 hours irradiation. Cellular viability decreased in the order QD LED > LED2 > LED4, as seen in the trypan blue results (Fig 2).

### Mitochondrial function is altered by the character of LED light in M-1 cells

**Fig 3:**
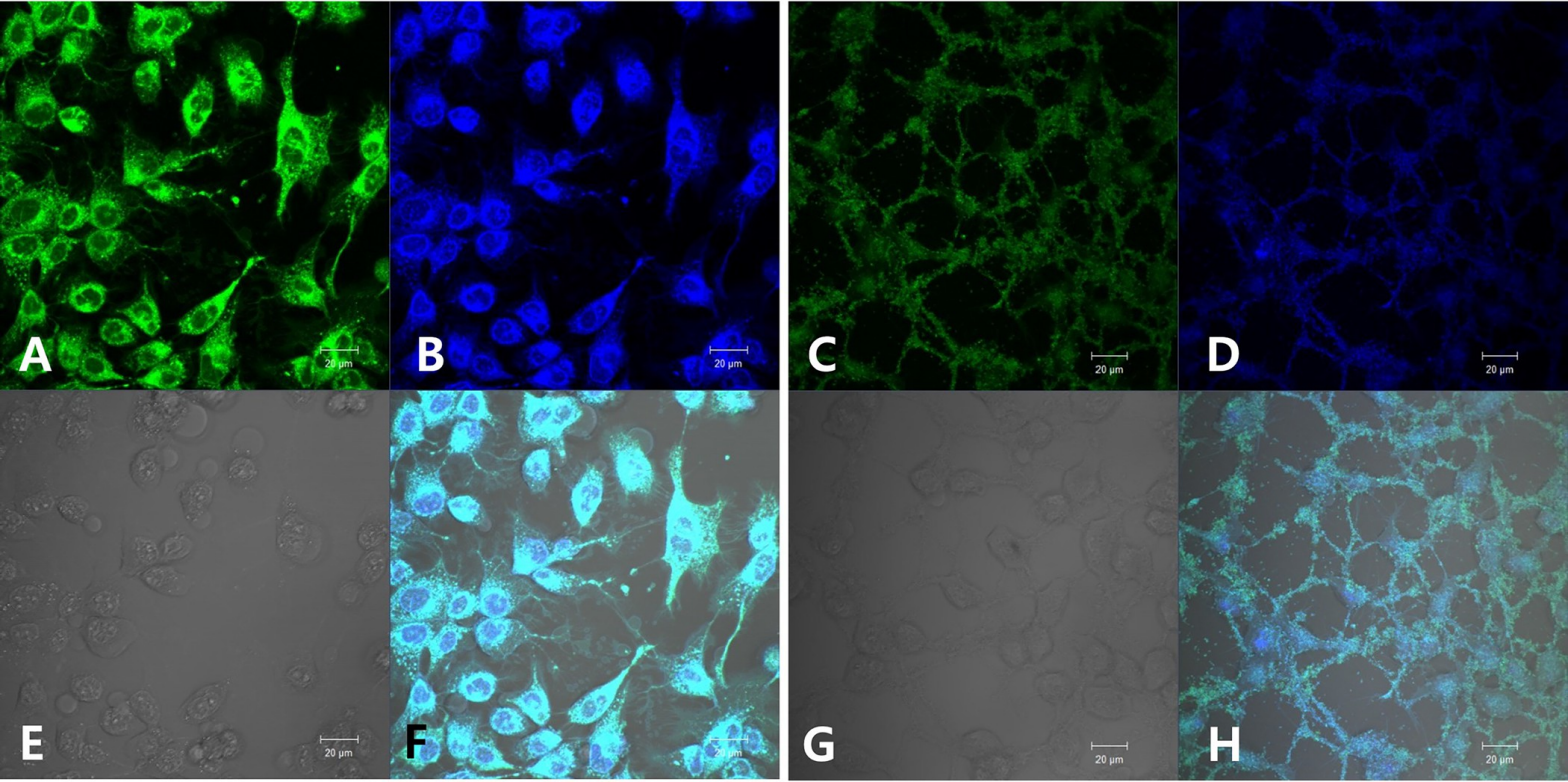
Morphological characteristic of M-1 cells compared to 293T cells and morphological characteristic of mouse brain CD1 cortical neuronal cells. Abundant mitochondria identified by immunostaining with MITO-ID green in M-1 cells (A,B,E,F) compared to relatively few mitochondria in 293T cells (green: mitochondria) (C,D,G,H). Typical characteristics of nerve cells, including nuclei and axons, were present in CD1 cells (I). A and C: mito-ID green detection stain; B and D: Hoechst33342 nuclear stain; E and G: Light microscope view; F and H: Merge image.(A,B,E,F images for M-1 cells and C,D,G,H images for 293T cells) (White scale bar: 20 μm, black scale bar: 50 μm).

We used M-1 cells, a mitochondria-rich mouse kidney collecting cell line that can be used to represent mitochondria-rich vertebrate cells [13], we confirmed this fact by comparing them with epithelial human embryonic kidney 293T cells by immunostaining with an MITO-ID green kit. Fluorescence microscopy revealed that there were more mitochondria in M-1 cells than in 293T cells (Fig 3A–3H).

**Fig 4:**
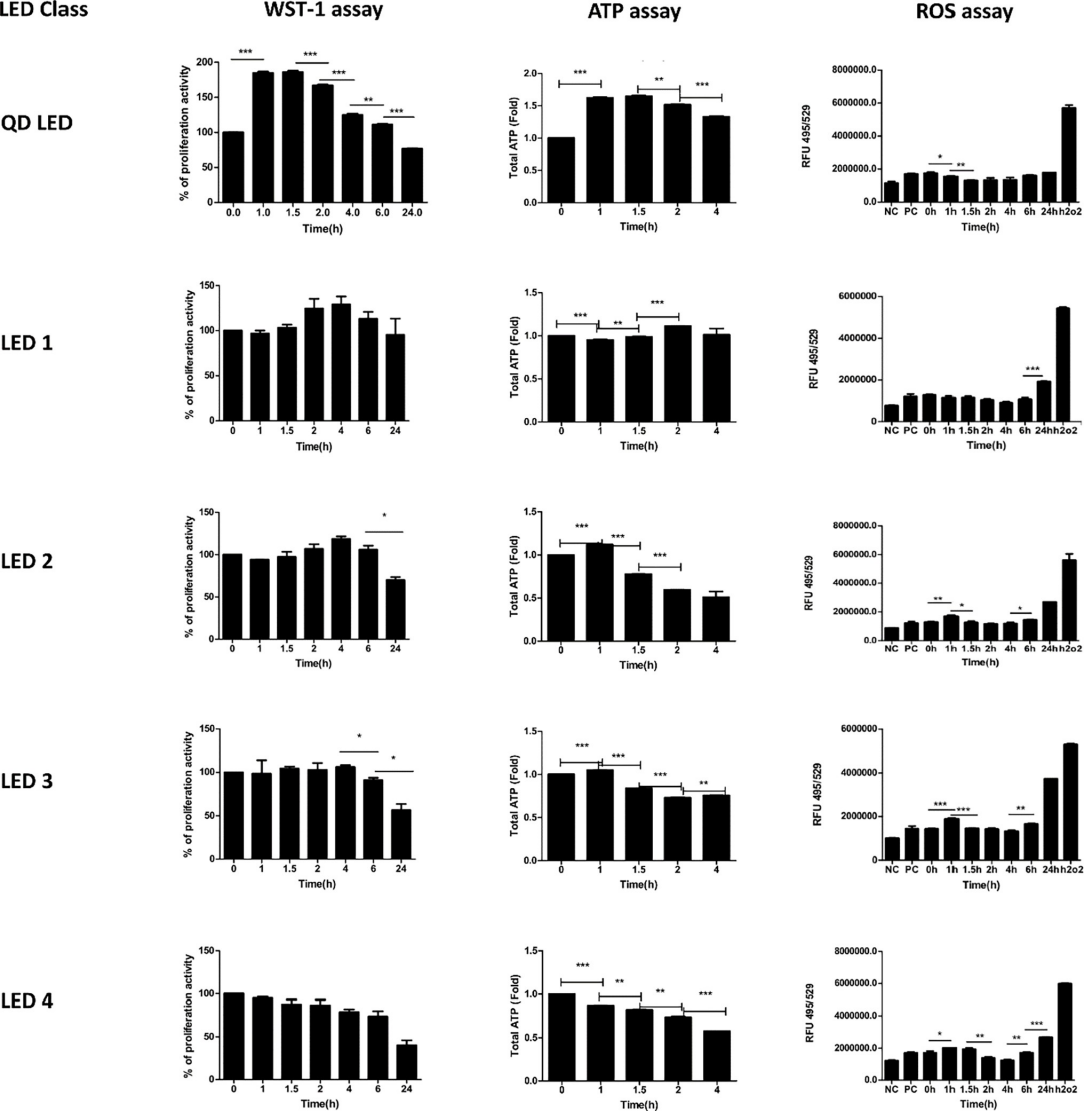
Mitochondrial functional assays in M-1 cells according to different LEDs and duration of application of LED light. Two types of mitochondrial functional assay (WST-1 and ROS level) and ATP assay were carried out with different classes of LED light (A) and for different periods of time in M-1 cells. The ATP assay was performed in quadrupicate and replicated at least three times using distinct cell harvests. Output was measured at 1, 1.5, 2, 4, 6, and 24 hours. (* p < 0.05, **p < 0.01, ***p<0.0001).

LED light exposure affected cellular proliferation according to results of WST- 1 assays (Fig 4). With exposure to QD LED light, cellular proliferation occurred from 1 to 6 hours and finally decreased compared to baseline (p<0.05). This proliferation occurred relatively earlier than it did with other LEDs. In the LED1 group, cellular proliferation occurred from 2 to 6 hours and also finally decreased compared to baseline (p<0.05). In the LED2 group, cellular proliferation was seen from 2 to 6 hours, as in LED1, however, the amount of proliferation was tiny. In the LED3 group, cellular proliferation was rare and proliferation decreased from 6 hours onwards, and there was no increase in cellular proliferation at the beginning of illumination and proliferation decreased gradually to the 24 hours point in the LED4 group.

Results of ATP assay were shown in Fig 4. In the QD LED, increasing ATP occurred from 1 to 4 hours (p<0.05). In the LED 1, amount of ATP were almost similar and slight increasing of ATP was shown at 2 hours (p<0.05). In the LED 2 & LED 3, there were slight elevation of ATP at 1 hour but after then amount of ATP decreased and, there was no increase in amount of ATP from beginning to end of illumination in the LED 4.

Changes in mitochondrial functioning which is represented by ROS were observed and corresponded well with the cellular proliferation results. In the QD LED and LED1 groups, there was no increased ROS generation from the beginning of illumination to 6 hours and finally increased at 24 hours; however, in the LED2, LED3, and LED4 groups, ROS generation increased starting at the beginning of illumination (p<0.05), even though there was little difference at 2 hours and 4 hours, and finally increased at 24 hours (Fig 4).

**Fig 5:**
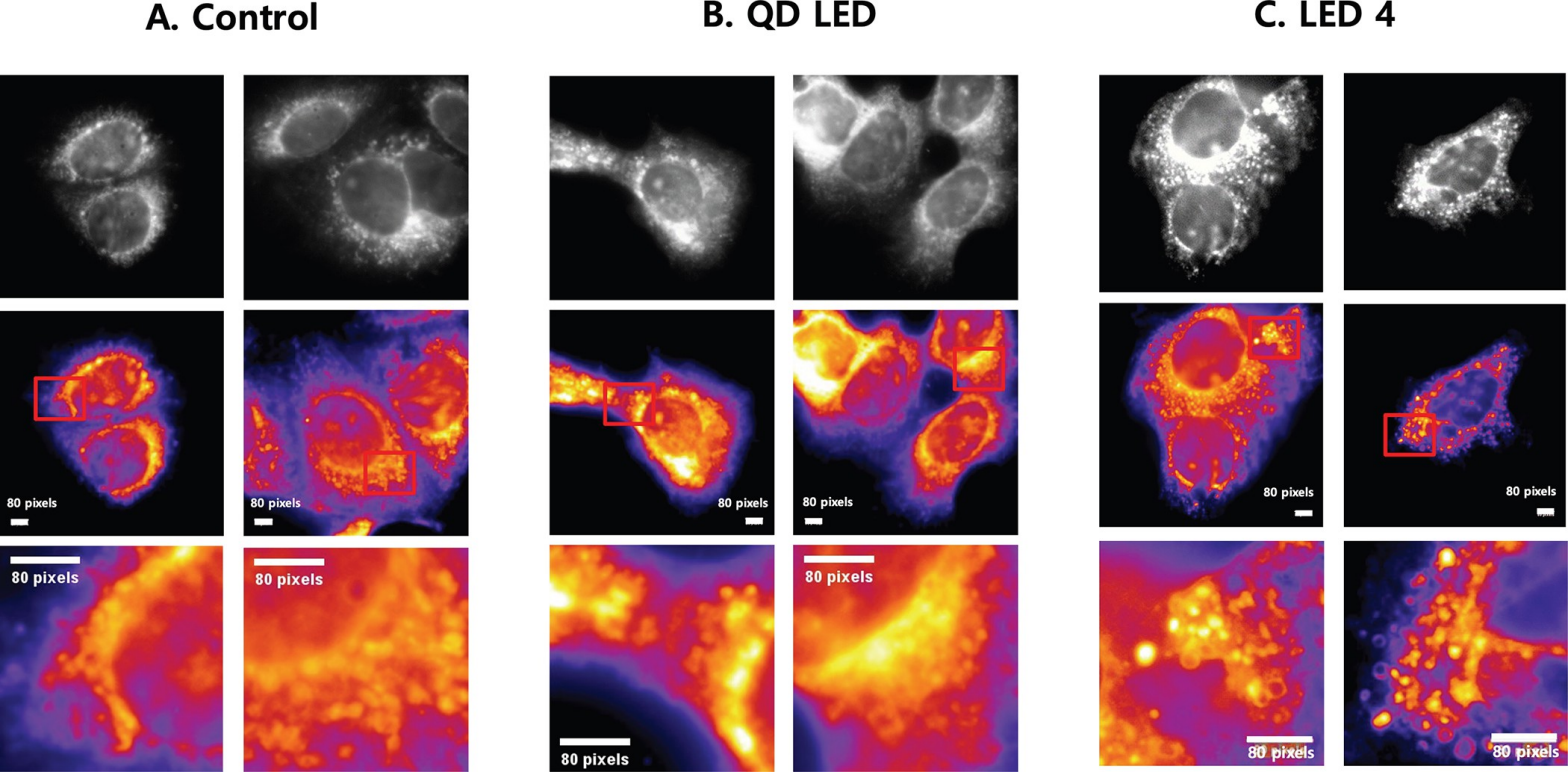
Mitochondrial morphology in M-1 cells according to control, QD LED and LED4. M-1 cells were exposed to control. (A), QD LED (B) and LED 4 (C) irradiation for 2 hours and mitochondrial morphology was observed using TIRF microscopy at 530 nm. The images were shown original mitochondria (upper panel), color coded image (middle panel) an zoom-in image (bottom panel) of red square at middle panel. Scale bar, 1 pixel: 160 nm.

Mitochondrial appearance image of control, QD LED and LED 4 groups were taken using TIRF at 530nm after 2 hours irradiation. Appearance of mitochondria between control and QD LED group were shown similar pattern, whereas the appearance of mitochondria of LED 4 were larger and grape like pattern which is typically shown in mitophagy (Fig 5).

### Cellular proliferation is altered by LED light in mouse brain CD1 cortex neurons

**Fig 6:**
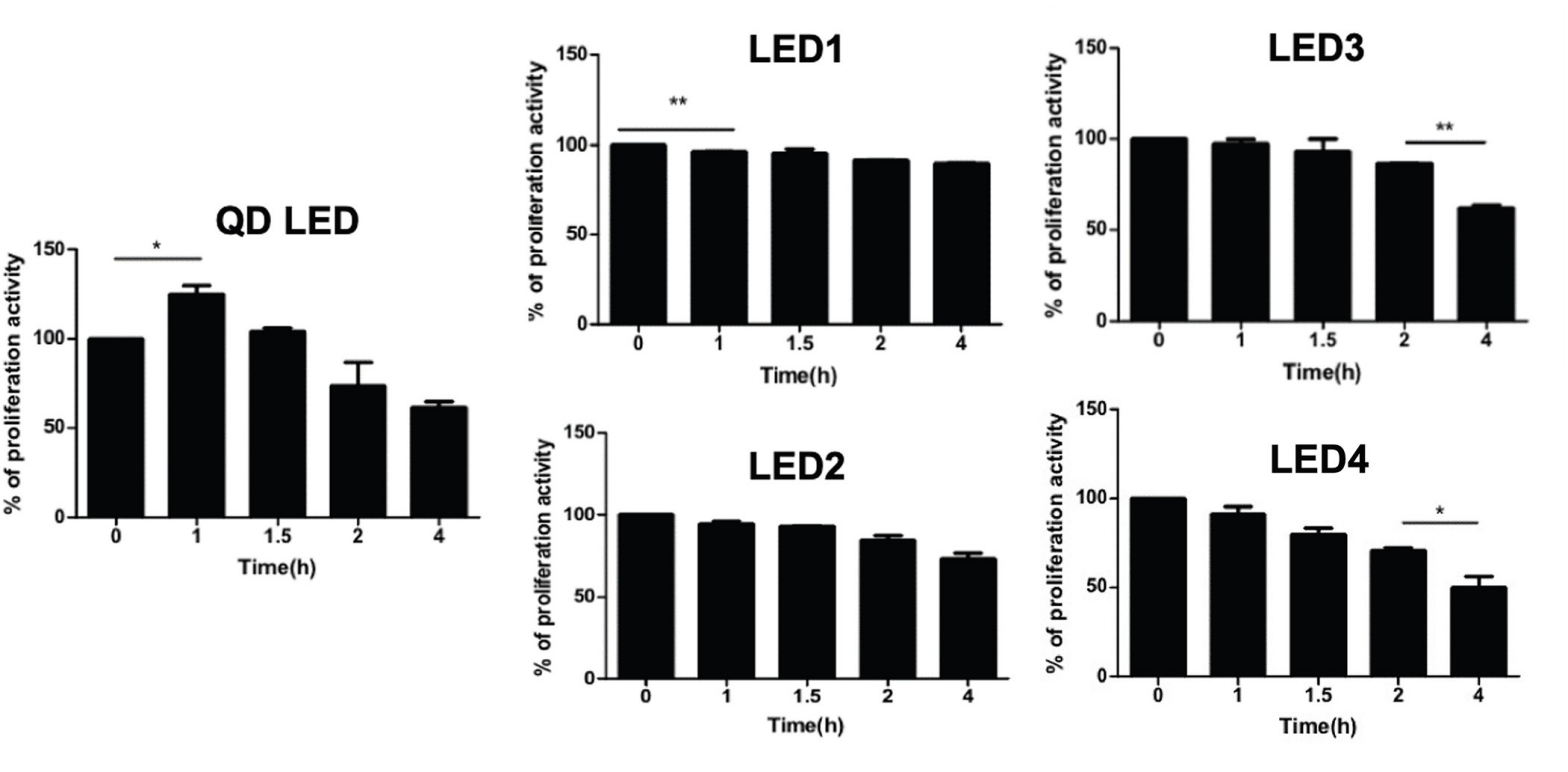
Mitochondrial activity assay in CD-1 cells according to LED type and duration of LED exposure. The WST-1 assay (B) was carried out in CD-1 cells. WST-1 was measured after 0 (baseline), 1, 1.5, 2, and 4 hours of LED illumination. (* p < 0.05, **p < 0.01).

Neuronal cells are more vulnerable than other cells in the same environment. Therefore, we measured cellular proliferation using the WST-1 assay for 4 hours in CD1 cortical neuronal cells exposed to different types of LED light. The results showed that cellular proliferation decreased with lower CRI values, higher CCT values, and longer duration of illumination and cellular proliferation was noticeably showed in QD LED and decreased cellular proliferation was showed in LED4 (Fig 6). These results were similar to the M-1 cell proliferation results.

## Discussion

Artificial LED lighting is becoming increasingly prevalent in the domestic lighting market because LEDs require low energy consumption and LEDs have benefit to change color easily. By 2016, the European Union had phased out traditional incandescent light sources from store shelves, with LED lighting emerging as the predominant choice for domestic illumination [12]. However, the distinctive spectral and energetic characteristics of white LED bulbs compared to other household lighting options have prompted concerns regarding their potential impact on human health, particularly on ocular safety [12]. The most prevalent LED lighting variant, the phosphor-conversion (PC) LED, employs a blue-light-emitting LED chip coated with a yellow phosphor layer to produce white light [16]. Despite the apparent normalcy of the resulting white light to human perception, a notable peak of blue light spanning from 460 to 500 nm is emitted within the white light spectrum, raising considerations about its potential effects [12, 17]. Therefore, some previous studies investigated the possibility that white LEDs could cause eye or retinal injury [12, 17]. These were mostly review articles that simply researched the relationship between white LED light and retinal injury. However, these days, LED lighting has been coming out in various generations [18] and the quality of an LED can be classified with CCT and CRI values, except factors involving luminous efficiency. Moreover, glaucoma patients have some trouble with visual impairment and low quality of life comparing to normal person [19–21] and glaucoma patient may have more time under artificial light, especially LED because of their visual impairment. Therefore, we wanted to investigate the relationship between LED quality and eye effect, especially RGC health indirectly using M-1 and CD-1 cells.

The CCT serves as a primary benchmark in assessing the tonal quality of white light, often characterizing it as either ’Hot’ or ’cold,’ evoking distinct sensory associations. Warm white illumination exudes a cozy ambiance with its subtle yellow-orange hue, typically manifesting with a CCT below 3500K. Conversely, cold white light tends towards cooler hues reminiscent of blue tones, featuring CCT ranges from 5500K upwards [12]. Therefore, a low CCT value means that the color put out by a light is near red and hot, whereas a high CCT value means that the color emitted is near blue and cold.

The CRI stands as another vital traditional performance metric for assessing color quality. It is a well-known color metric that is used to reproduce highly saturated colors of objects illuminated by a white light [18]. A superior white light source should faithfully render the true colors of its illuminated surroundings, a critical aspect particularly in indoor lighting applications. Furthermore, research by Raynham et al. suggests that adequate color rendition enhances road safety by improving color contrast, particularly in low ambient lighting scenarios such as outdoor settings [22]. Originating from the Commission Internationale de l’Eclairage (CIE) in 1971 [23], the CRI underwent refinement to its present iteration in 1995 [24]. With a maximum value of 100 signifying optimal color rendering and -100 representing the poorest rendition, the CRI serves as a pivotal tool for evaluating lighting quality [25]. By definition, daylight has a CRI of 100, therefore, the main goal of artificial lighting is achievement of higher CRI and lower CCT values. Scientists and engineers who work on LED lighting have tried to invent high-CRI LEDs with good luminous efficacy [18, 26].

The major finding of our study was that cultured cells exposed to higher CRI and lower CCT LED lights exhibited more normal mitochondrial function as assessed by cellular proliferation and ATPase activity, more balanced and low ROS production, and less cell death than cells exposed to lower CRI and higher CCT lights. A lower CCT value means that light is near red in wavelength and a higher CRI value means that the character of the light is similar to that of sunlight and has a higher proportion of red waves, which is true for traditional incandescent lighting. Shang et al also showed similar results. They revealed that white LED at domestic lighting level which have more blue light can induce photochemical injury of retina [17]. Although we did not use RGCs in this study, it seems to be suggested that high-quality LED lighting may induce normal mitochondrial functions and less cellular toxicity in RGCs, because we found similar cellular results from M-1 cell and CD1 cortex neuronal cells which were cells with two typical characteristics of RGCs.

The presence of high concentration of ROS can overpower the cell’s innate defense systems, potentially leading to various outcomes such as the generation of internal antioxidants [27] and the initiation of pathways associated with programmed cell death. One pivotal role of ROS lies in mediating vital cellular functions like apoptosis, which holds significance in maintaining cellular balance as well as in numerous pathological conditions [28]. Numerous studies have demonstrated apoptosis as the primary mechanism underlying Retinal Ganglion Cell (RGC) death, with its activation stemming from various processes. The most important organelle in controlling ROS and oxidative stress is the mitochondria.

Mitochondria are cytoplasmic organelles that regulate both metabolic and apoptotic signaling pathways. Their major functions include generating energy in the form of adenosine triphosphate (ATP), regulating cellular calcium homeostasis, balancing ROS production and detoxification, mediating apoptotic cell death, and carrying out synthesis and metabolism of various key molecules such as fatty acids [29–31]. Mitochondrial dysfunction leads to diverse pathologies in a multitude of human disease states [32].

The quantity of mitochondria within cells fluctuates based on the metabolic requirements of those cells. Mitochondria-rich cells have more mitochondria than do adjacent cells from the same epithelium; such cells include kidney collecting duct cells in vertebrates, amphibian urinary bladder cells, and amphibian epidermal cells [14]. Moreover, central nervous system (CNS) neurons require abundant mitochondria; approximately 90% of the ATP generated by mitochondria is dedicated to sustaining the intricate membrane dynamics crucial for the propagation of action potentials and the survival of neurons. Notably, within the retina, unmyelinated Retinal Ganglion Cell (RGC) axons harbor a particularly abundant population of mitochondria, emphasizing their pivotal role in supporting neuronal function and viability [33–35] and in the inner segments of photoreceptors [36]. Mitochondrial density can be changed according to local energy demands. In the unmyelinated prelaminar and laminar regions of the optic nerve, where the maintenance of membrane potential demands significant energy expenditure, a notable abundance of voltage-gated Na+ channels is observed [37]. Consequently, this region exhibits the highest concentration of mitochondria, alongside structural cytoskeleton proteins, reflecting their pivotal role in energy metabolism, with a significant decrease in their concentration occurring more posteriorly with optic nerve myelination [38].

There are several crucial risk factors of mitochondrial dysfunction. Aging, genetics, increased IOP, ischemia, and light are the most common factors associated with mitochondrial dysfunction [29, 39, 40]. Light of different wavelengths interacts differently with the four primary mitochondrial protein complexes responsible for ATP generation. Blue light potentially has a negative influence on mitochondria in RGCs. Exposure to blue light can lead to a reduction in mitochondrial dehydrogenase activity and a decline in redox potential. This disruption results in decreased ATP production and elevated levels of ROS, both of which are indicative of oxidative stress. This may be of significance in glaucoma, where it’s probable that the mitochondria of RGCs are already compromised, rendering them more vulnerable to the effects of blue light. Long–wavelength or red light influences mitochondrial complex IV, also known as cytochrome oxidase, enhancing ATP production rates and mitigating ROS levels. This process yields several advantageous factors [9, 31, 41, 42]. Moreover, damaged mitochondria are selectively turned over in eukaryotic cells via mitophagy, autophagosome engulfs to damaged mitochondria and appearance of mitochondria are changed larger and grape-like pattern [43, 44]. These results suggest that LED light that includes more red and near-infrared wavelengths and that is similar to natural sunlight, which is represented by high CRI and low CCT values, has positive effects on mitochondrial physiology and cellular viability.

Another interesting finding is that the wavelength and the spectral power distributions (SPDs) of light may be important for mitochondrial function and may represent crucial risk factors that contribute to photochemical retinal injury. When we compared QD LED and LED 1 light, we noticed that many specifications were similar between the two except for the amount of near-infrared light and Lux (luminous flux received by a surface)(Fig 2). QD LED light had more near-infrared wavelengths and less Lux. Cells exposed to QD LED light had more cellular activity, ATP level and less ROS than did those exposed to LED1 light, although cells exposed to LED1 light had elevated cellular activity and low ROS generation compared to the other LED lights. One possible reason for this finding concerns SPDs, and another is that light is a particle [45]. In the same situation, more focal near-infrared wavelength light from an LED may be beneficial to mitochondrial physiological functioning and an excessive number of particles of light may have a negative effect on the mitochondria, especially near-blue wave light. Shang et al. also noted that SPDs and wavelength were very important to cell survival in vivo [17].

It is very difficult to make LED lights that fit the above conditions, such as narrow focal wavelength and low Lux. However, QD lights, a third-generation LED technology, makes it relatively easy. This is because QD LEDs are capable of fine spectral tuning via by size control and narrow-band emissions [25].

A final interesting finding of our study is that the duration of light irradiation is important. According to our results, after 24 hours of LED irradiation, ROS activation and associated cell death were increased in all groups regardless of the type of LED used. This is very important nowadays because many people remain at work for a long time, some people work more at night time, and personal computers, smartphones, tablet PCs, etc. are used by a very large number of people. As time goes on, the amount of time that we all spend under artificial lighting such as LED lighting will continue to increase. Therefore, the cumulative time that we are exposed to light irradiation will increase, so the chance of cellular damage will also increase. Therefore, it is essential that we further investigate the safe duration of usage, intensity, and characteristics of artificial light, especially LED light. Moreover, our study may be the first step for safety mandates concerning artificial light usage.

A limitation of this study is that we did not use primary RGCs cells. This was because, as mentioned previously, primary cultured RGCs cells cannot survive in our study conditions, such as the long time in cell culture and long exposure to LED irradiation like in real world conditions. Thus, we used M-1 cells and CD-1 cells, which represented 2 major characteristics of RGCs. Finally, it is necessary to confirm our results in an in vivo study including a retinal explant model [46].

## Conclusion

Glaucoma has been associated with the function of an important cytoplasmic organelle, the mitochondrion, which could be affected by light. High-quality LEDs used for an adequate amount of time could improve physiological mitochondrial function and reduce cellular damage. Therefore, further investigation into high-quality LED lighting and a better understanding of the properties of LEDs may facilitate the development of novel neuroprotective/regenerative therapeutic strategies for common, difficult to treat CNS neurodegenerative disease states, as well as conditions that result from mitochondrial dysfunction related to oxidative stress in addition to glaucoma.

## References

[1] Quigley HA, Broman AT. The number of people with glaucoma worldwide in 2010 and 2020. Br J Ophthalmol. 2006;90(3): 262–267. doi: 10.1136/bjo.2005.081224 16488940 PMC1856963

[2] Causes of blindness and vision impairment in 2020 and trends over 30 years, and prevalence of avoidable blindness in relation to VISION 2020: the Right to Sight: an analysis for the Global Burden of Disease Study. Lancet Glob Health. 2021;9(2): e144–e160. doi: 10.1016/s2214-109x(20)30489-7 33275949 PMC7820391

[3] Quigley HA. Ganglion cell death in glaucoma: pathology recapitulates ontogeny. Aust N Z J Ophthalmol. 1995;23(2): 85–91. doi: 10.1111/j.1442-9071.1995.tb00135.x 7546696

[4] Baloyannis SJ, Costa V, Michmizos D. Mitochondrial alterations in Alzheimer’s disease. Am J Alzheimers Dis Other Demen. 2004;19(2): 89–93. doi: 10.1177/153331750401900205 15106389 PMC10834007

[5] Mizuno Y, Ikebe S, Hattori N, Nakagawa-Hattori Y, Mochizuki H, Tanaka M, et al. Role of mitochondria in the etiology and pathogenesis of Parkinson’s disease. Biochim Biophys Acta. 1995;1271(1): 265–274. doi: 10.1016/0925-4439(95)00038-6 7599219

[6] Shigenaga MK, Hagen TM, Ames BN. Oxidative damage and mitochondrial decay in aging. Proc Natl Acad Sci U S A. 1994;91(23): 10771–10778. doi: 10.1073/pnas.91.23.10771 7971961 PMC45108

[7] Trimmer PA, Swerdlow RH, Parks JK, Keeney P, Bennett JP, Jr., Miller SW, et al. Abnormal mitochondrial morphology in sporadic Parkinson’s and Alzheimer’s disease cybrid cell lines. Exp Neurol. 2000;162(1): 37–50. doi: 10.1006/exnr.2000.7333 10716887

[8] Williams PA, Harder JM, Foxworth NE, Cochran KE, Philip VM, Porciatti V, et al. Vitamin B3 modulates mitochondrial vulnerability and prevents glaucoma in aged mice. Science. 2017;355(6326): 756–760. doi: 10.1126/science.aal0092 28209901 PMC5408298

[9] Osborne NN, Nunez-Alvarez C, Del Olmo-Aguado S, Merrayo-Lloves J. Visual light effects on mitochondria: The potential implications in relation to glaucoma. Mitochondrion. 2017;36: 29–35. doi: 10.1016/j.mito.2016.11.009 27890822

[10] Osborne NN, Lascaratos G, Bron AJ, Chidlow G, Wood JP. A hypothesis to suggest that light is a risk factor in glaucoma and the mitochondrial optic neuropathies. Br J Ophthalmol. 2006;90(2): 237–241. doi: 10.1136/bjo.2005.082230 16424541 PMC1860161

[11] Chepesiuk R. Missing the dark: health effects of light pollution. Environ Health Perspect. 2009;117(1): A20–27. doi: 10.1289/ehp.117-a20 19165374 PMC2627884

[12] Behar-Cohen F, Martinsons C, Vienot F, Zissis G, Barlier-Salsi A, Cesarini JP, et al. Light-emitting diodes (LED) for domestic lighting: any risks for the eye? Prog Retin Eye Res. 2011;30(4): 239–257. doi: 10.1016/j.preteyeres.2011.04.002 21600300

[13] Hong S, Iizuka Y, Kim CY, Seong GJ. Isolation of primary mouse retinal ganglion cells using immunopanning-magnetic separation. Mol Vis. 2012;18: 2922–2930. https://www.ncbi.nlm.nih.gov/pubmed/23233794 23233794 PMC3519380

[14] Brown D, Breton S. Mitochondria-rich, proton-secreting epithelial cells. J Exp Biol. 1996;199(Pt 11): 2345–2358. doi: 10.1242/jeb.199.11.2345 9114500

[15] Mattheyses AL, Simon SM, Rappoport JZ. Imaging with total internal reflection fluorescence microscopy for the cell biologist. J Cell Sci. 2010;123(Pt 21): 3621–3628. doi: 10.1242/jcs.056218 20971701 PMC2964103

[16] Spivey A. The mixed blessing of phosphor-based white LED. Environ Health Perspect. 2011;119(11): A472–473. doi: 10.1289/ehp.119-a472 22171376 PMC3226516

[17] Shang YM, Wang GS, Sliney D, Yang CH, Lee LL. White light-emitting diodes (LEDs) at domestic lighting levels and retinal injury in a rat model. Environ Health Perspect. 2014;122(3): 269–276. doi: 10.1289/ehp.1307294 24362357 PMC3948037

[18] Yoon HC, Oh JH, Lee S, Park JB, Do YR. Circadian-tunable perovskite quantum dot-based down-converted multi-package white LED with a color fidelity index over 90. Sci Rep. 2017;7(1): 2808. doi: 10.1038/s41598-017-03063-7 28584229 PMC5459832

[19] Alqudah A, Mansberger SL, Gardiner SK, Demirel S. Vision-related quality of life in glaucoma suspect or early glaucoma patients. J Glaucoma. 2016;25(8): 629–633. doi: 10.1097/IJG.0000000000000445 27483331 PMC4975427

[20] Gupta V, Srinivasan G, Mei SS, Gazzard G, Sihota R, Kapoor KS. Utility values among glaucoma patients: an impact on the quality of life. Br J Ophthalmol. 2005;89(10): 1241–1244. doi: 10.1136/bjo.2005.068858 16170108 PMC1772859

[21] Wu P, Xi S, Xia H, Lu H, Guo W. Survey on vision-related quality of life and self-management among patients with glaucoma. J Glaucoma. 2014;23(2): 75–80. doi: 10.1097/IJG.0b013e318265bbf3 22936279

[22] Raynham P, Saksvikronning T. White light and facial recognition. The Lighting Journal. 2003;68(1): 29–33. https://www.igov.nl/images/stories/content/documenten/raynham_20white_20light0001.pdf

[23] Robertson AR. Colorimetry. Reports on Progress in Physics. 1978;41(4): 469–510. doi: 10.1088/0034-4885/41/4/001

[24] Method of measuring and specifying colour rendering properties of light sources, CIE publication 13.3–1995. Color Res Appl. 1995;20(3): 212. doi: 10.1002/col.5080200313

[25] Erdem T, Demir HV. Color science of nanocrystal quantum dots for lighting and displays. Nanophotonics. 2013;2(1): 57–81. doi: 10.1515/nanoph-2012-0031

[26] Tu CC, Hoo JH, Bohringer KF, Lin LY, Cao G. Red-emitting silicon quantum dot phosphors in warm white LEDs with excellent color rendering. Opt Express. 2014;22 Suppl 2: A276–281. doi: 10.1364/OE.22.00A276 24922236

[27] Di Marco E, Jha JC, Sharma A, Wilkinson-Berka JL, Jandeleit-Dahm KA, de Haan JB. Are reactive oxygen species still the basis for diabetic complications? Clin Sci (Lond). 2015;129(2): 199–216. doi: 10.1042/CS20150093 25927680

[28] Nickells RW, Zack DJ. Apoptosis in ocular disease: a molecular overview. Ophthalmic Genet. 1996;17(4): 145–165. doi: 10.3109/13816819609057889 9010866

[29] Chan DC. Mitochondria: dynamic organelles in disease, aging, and development. Cell. 2006;125(7): 1241–1252. doi: 10.1016/j.cell.2006.06.010 16814712

[30] Schon EA, Manfredi G. Neuronal degeneration and mitochondrial dysfunction. J Clin Invest. 2003;111(3): 303–312. doi: 10.1172/JCI17741 12569152 PMC151870

[31] Osborne NN, Nunez-Alvarez C, Del Olmo-Aguado S. The effect of visual blue light on mitochondrial function associated with retinal ganglions cells. Exp Eye Res. 2014;128: 8–14. doi: 10.1016/j.exer.2014.08.012 25193034

[32] Liang C, Ahmad K, Sue CM. The broadening spectrum of mitochondrial disease: shifts in the diagnostic paradigm. Biochim Biophys Acta. 2014;1840(4): 1360–1367. doi: 10.1016/j.bbagen.2013.10.040 24239706

[33] Bristow EA, Griffiths PG, Andrews RM, Johnson MA, Turnbull DM. The distribution of mitochondrial activity in relation to optic nerve structure. Arch Ophthalmol. 2002;120(6): 791–796. doi: 10.1001/archopht.120.6.791 12049585

[34] Carelli V, La Morgia C, Valentino ML, Rizzo G, Carbonelli M, De Negri AM, et al. Idebenone treatment in Leber’s hereditary optic neuropathy. Brain. 2011;134(Pt 9): e188. doi: 10.1093/brain/awr180 21810891

[35] Wang L, Dong J, Cull G, Fortune B, Cioffi GA. Varicosities of intraretinal ganglion cell axons in human and nonhuman primates. Invest Ophthalmol Vis Sci. 2003;44(1): 2–9. doi: 10.1167/iovs.02-0333 12506048

[36] Stone J, van Driel D, Valter K, Rees S, Provis J. The locations of mitochondria in mammalian photoreceptors: relation to retinal vasculature. Brain Res. 2008;1189: 58–69. doi: 10.1016/j.brainres.2007.10.083 18048005

[37] Barron MJ, Griffiths P, Turnbull DM, Bates D, Nichols P. The distributions of mitochondria and sodium channels reflect the specific energy requirements and conduction properties of the human optic nerve head. Br J Ophthalmol. 2004;88(2): 286–290. doi: 10.1136/bjo.2003.027664 14736793 PMC1771975

[38] Balaratnasingam C, Morgan WH, Johnstone V, Cringle SJ, Yu DY. Heterogeneous distribution of axonal cytoskeleton proteins in the human optic nerve. Invest Ophthalmol Vis Sci. 2009;50(6): 2824–2838. doi: 10.1167/iovs.08-3206 19168905

[39] Lascaratos G, Garway-Heath DF, Willoughby CE, Chau KY, Schapira AH. Mitochondrial dysfunction in glaucoma: understanding genetic influences. Mitochondrion. 2012;12(2): 202–212. doi: 10.1016/j.mito.2011.11.004 22138560

[40] Osborne NN, Casson RJ, Wood JP, Chidlow G, Graham M, Melena J. Retinal ischemia: mechanisms of damage and potential therapeutic strategies. Prog Retin Eye Res. 2004;23(1): 91–147. doi: 10.1016/j.preteyeres.2003.12.001 14766318

[41] Godley BF, Shamsi FA, Liang FQ, Jarrett SG, Davies S, Boulton M. Blue light induces mitochondrial DNA damage and free radical production in epithelial cells. J Biol Chem. 2005;280(22): 21061–21066. doi: 10.1074/jbc.M502194200 15797866

[42] Tribble JR, Otmani A, Sun S, Ellis SA, Cimaglia G, Vohra R, et al. Nicotinamide provides neuroprotection in glaucoma by protecting against mitochondrial and metabolic dysfunction. Redox Biol. 2021;43: 101988. doi: 10.1016/j.redox.2021.101988 33932867 PMC8103000

[43] Hsieh CW, Yang WY. Omegasome-proximal PtdIns(4,5)P2 couples F-actin mediated mitoaggregate disassembly with autophagosome formation during mitophagy. Nat Commun. 2019;10(1): 969. doi: 10.1038/s41467-019-08924-5 30814505 PMC6393429

[44] Wong YC, Holzbaur EL. Optineurin is an autophagy receptor for damaged mitochondria in parkin-mediated mitophagy that is disrupted by an ALS-linked mutation. Proc Natl Acad Sci U S A. 2014;111(42): E4439–4448. doi: 10.1073/pnas.1405752111 25294927 PMC4210283

[45] Anglin J. Quantum optics: particles of light. Nature. 2010;468(7323): 517–518. doi: 10.1038/468517a 21107419

[46] Kim M, Kim JY, Rhim WK, Cimaglia G, Want A, Morgan JE, et al. Extracellular vesicle encapsulated nicotinamide delivered via a trans-scleral route provides retinal ganglion cell neuroprotection. Acta Neuropathol Commun. 2024;12(1): 65. doi: 10.1186/s40478-024-01777-0 38649962 PMC11036688

