# Correction: The effects of light emitting diodes on mitochondrial function and cellular viability of M-1 cell and mouse CD1 brain cortex neurons

**DOI:** 10.1371/journal.pone.0325398

**Published:** 2025-05-28

**Authors:** Jong Soo Lee, Hyun Jin Park, Sang Ook Kang, Sang Hak Lee, Chang Kyu Lee

The authors Sang Hak Lee and Chang Kyu Lee should not have been attributed equal contribution but as co-corresponding authors of this work. Sang Hak Lee’s email address is: shlee@pusan.ac.kr.
